# Atlas of receptor genes expressed by the bovine morula and corresponding ligand‐related genes expressed by uterine endometrium

**DOI:** 10.1002/mrd.23534

**Published:** 2021-10-01

**Authors:** Lei Sang, Yao Xiao, Zongliang Jiang, Niamh Forde, Xiuchun Cindy Tian, Patrick Lonergan, Peter J. Hansen

**Affiliations:** ^1^ Institute of Animal Husbandry and Veterinary Medicine Fujian Academy of Agricultural Sciences Fuzhou Fujian China; ^2^ Department of Animal Sciences, D.H. Barron Reproductive and Perinatal Biology Research Program, and Genetics Institute University of Florida Gainesville Florida USA; ^3^ Institute of Animal Science and Veterinary Medicine Shandong Academy of Agricultural Sciences Jinan Shandong China; ^4^ School of Animal Sciences, AgCenter Louisiana State University Baton Rouge Louisiana USA; ^5^ Department of Discovery and Translational Sciences University of Leeds Leeds UK; ^6^ Department of Animal Science University of Connecticut Storrs Connecticut USA; ^7^ School of Agriculture and Food Science University College Dublin Ireland

**Keywords:** cell signaling, embryo, endometrium, morula, receptors

## Abstract

Regulation of the mammalian embryo involves cell‐signaling molecules produced by the maternal oviduct and endometrium. Here, datasets on the transcriptome of the gestational Days 5 and 6 bovine morula and Day 5 maternal endometrium were examined to identify receptor genes expressed by the morula and expression of the corresponding ligand‐related genes in the endometrium. A total of 175 receptor genes were identified in the morula, including 48 encoding for growth factors or WNT signaling molecules, 25 for cytokines and chemokines, 35 involved in juxtacrine and matricellular signaling and 25 encoding for receptors for small molecules. Some of the highly‐expressed pairs of endometrial ligand and embryo receptor genes included *MDK* and its receptors *ITGB1, SDC4* and *LRP2*, *WNT5A* (*RYK*), *VEGFA* (*ITGB1), GPI* (*AMFR*), and the hedgehog proteins *IHH* and *DHH* (*HHIP*). The most highly expressed receptors for small molecules were *GPRC5C* (retinoic acid receptor), *PGRMC1* (progesterone), and *CHRNB2* (acetylcholine). There were also 84 genes encoding for cell signaling ligands expressed by the morula, with the most highly expressed being *GPI, AIMP1, TIMP1, IK*, and *CCN2*. The atlas of receptor and ligand genes should prove useful for understanding details of the communication between the embryo and mother that underlies optimal embryonic development.

## 1 INTRODUCTION

The mammalian embryo exhibits a great deal of autonomy in control of its development—embryos can develop from the zygote to blastocyst stage of development in a defined culture medium composed of salts, energy source, amino acids, and a surfactant (Block et al., 2009). Autonomy is not absolute, however. Embryos produced in vitro differ in biochemical, molecular, and ultrastructural characteristics from those developing in vivo (Hansen, 2020). Furthermore, normal development beyond the hatched blastocyst stage, when the trophoblast of the ruminant embryo undergoes rapid elongation, has not been recapitulated in vitro and requires exposure to the uterine environment to occur (Sánchez et al., 2019).

The physiology and development of the early embryo is regulated by cell‐signaling molecules called embryokines that are produced by the oviduct and endometrium. In the cow, the focus of the current investigation, these molecules include IGF1 (Bonilla et al., 2011; Sirisathien et al., 2003), CSF2 (Loureiro et al., 2009), the WNT inhibitor DKK1 (Denicol et al., 2014), activin A (Kannampuzha‐Francis et al., 2017) and IL6 (Wooldridge et al., 2019). Among the embryo functions regulated by embryokines are promotion of development to the blastocyst stage (Bonilla et al., 2011; Kannampuzha‐Francis et al., 2017), protection from cell stress (Jousan & Hansen, 2007), and control of lineage commitment towards inner cell mass and trophectoderm (Denicol et al., 2014; Sang et al., 2020; Wooldridge et al., 2019). Some embryokines, such as CSF2 (Kannampuzha‐Francis et al., 2015) and DKK1 (Tríbulo et al., 2017), have been reported to program development in a way that affects postnatal phenotype.

One limitation to understanding the molecular basis for embryo‐maternal cross talk in the preimplantation period is insufficient information regarding the array of cell‐signaling receptors expressed by the early embryo. Others have interrogated ligand‐receptor interactions between the bovine conceptus and associated endometrium around the critical window of maternal recognition of pregnancy (Mamo et al., 2012). However, data on such interactions at earlier stages are lacking. Here we used datasets on the transcriptome of the gestational Days 5 and 6 bovine morula and Day 5 maternal endometrium to identify receptor genes expressed by the morula and determine which of these receptor genes was accompanied by expression of the corresponding ligand‐related genes in the endometrium. The rationale was that the embryo is likely to be regulated by cell‐signaling molecules for which receptor expression is high and the ligand is produced by the endometrium. Analysis was performed for tissues collected on Days 5–6 of gestation because this stage follows the major activation of the embryonic genome at the 8‐cell stage (Jiang et al., 2014), coincides with the transit of the bovine embryo from the oviduct to the uterine lumen at Day 4 or 5 after insemination (Hackett et al., 1993) and immediately precedes formation of the blastocyst (Betteridge & Fléchon, 1988). Results revealed a plethora of cell‐signaling systems including many that were hitherto not implicated in regulation of function of the preimplantation embryo.

## 2 RESULTS

### 2.1 Overall number of embryo receptor genes

**Figure 1:**
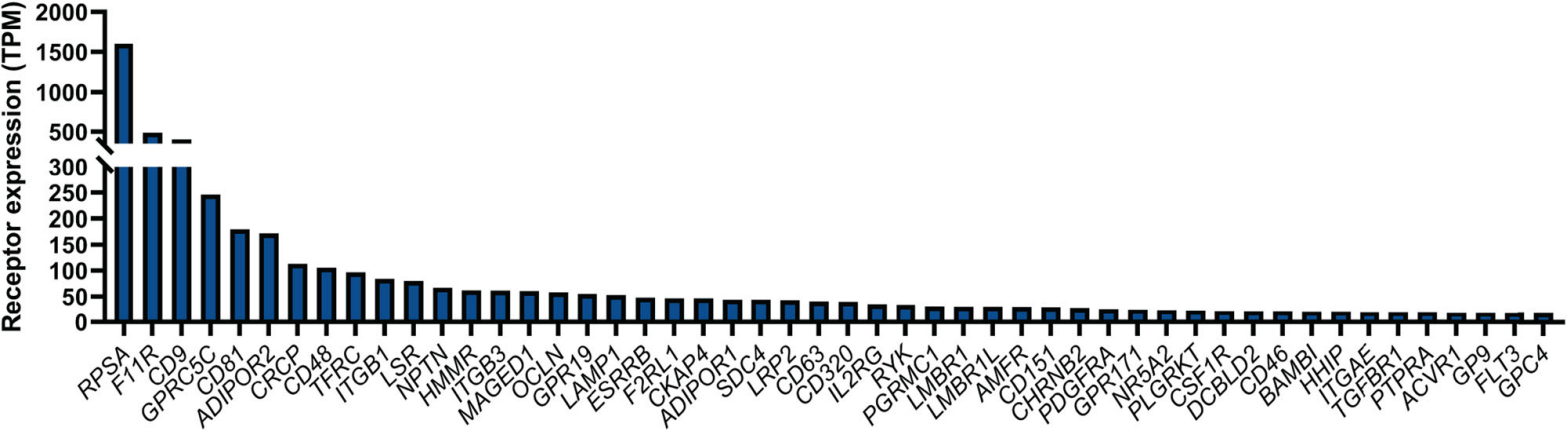
Transcript abundance for the 50 most highly‐expressed receptor genes for the bovine morula. Results are the average of 4 embryos

Screening of the transcriptome of the Days 5 and 6 morula identified a total of 175 receptor genes in which expression was >2 transcripts per kilobase million (TPM). Many receptors have multiple ligands, which in some cases are not involved in cell signaling. Details on each receptor, including transcript abundance of the receptor gene in the morula and endometrial expression of either ligand genes (for protein ligands) or enzymes involved in synthesis of the ligand (for select nonprotein ligands), are shown in Supporting Information File S1, Table S1. Transcript abundance for the 50 highest expressed receptor genes is shown in Figure 1. A total of 8 receptor genes had very high transcript abundance (>100 TPM). Of these, the three most highly expressed receptor genes were *RPSA*, which functions as a laminin receptor, *F11R*, involved in tight junction assembly, and *CD9*, which binds the disintegrin and metalloproteinase domain protein ADAM2 and heparin‐binding epidermal growth factor (HBEGF). Other receptor genes in which TPM was >100 were the G‐protein coupled receptor *GPRC5C*, which is a retinoic acid receptor; *CD81*, which binds complement C3 and the cell surface proteoglycan GPC3; the adiponectin receptor *ADIPOR2;* the calcitonin receptor component *CRCP;* and the IL18 receptor *CD48*.

Analysis of the receptor genes expressed in the morula by Ingenuity Pathway Analysis identified 103 canonical pathways in which there was overrepresentation (*p* < 0.05) of genes (Supporting Information File S1, Table S2). Among the pathways with at least 10 genes was phagosome formation (*n* = 44 genes), CREB signaling in neurons (*n* = 43 genes), STAT3 pathway (*n* = 16 genes), PTEN signaling (*n* = 15), PI3K/AKT signaling (*n* = 15 genes), WNT/β‐catenin signaling (*n* = 14 genes), and human embryonic stem cell pluripotency (*n* = 10 genes).

### 2.2 Genes encoding receptors for growth factors and WNT ligands

Subsequent analyses of receptor gene expression in the morula‐stage embryo were conducted for specific classes of ligands to identify receptor‐ligand pairs where expression of the receptor gene in the morula and of the ligand gene in the endometrium were high. It was reasoned that these receptor‐ligand signaling systems would be likely to be involved in regulation of embryonic function. Genes were considered highly expressed if TPM >10 (i.e., logTPM >1).

**Figure 2:**
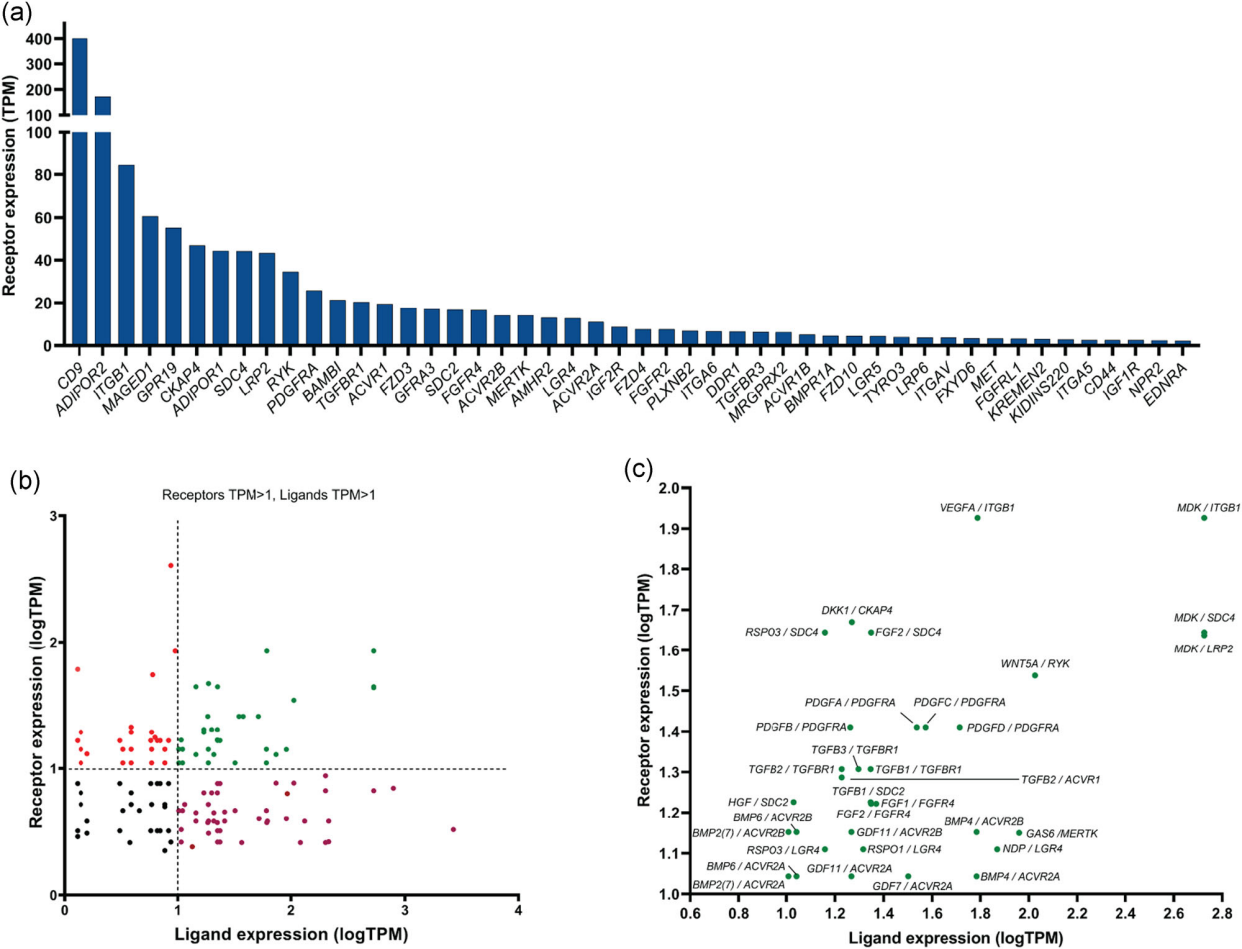
Relationship between the magnitude of expression by the bovine morula of genes encoding for receptors for growth factors or WNT ligands and expression of the corresponding ligand genes in the endometrium. (a) Transcript abundance for the 48 genes expressed by the morula that were characterized as encoding for receptors for growth factors or WNT ligands. Results are the average of 4 embryos. (BC) Association between expression of individual receptor genes in the morula (y‐axis) and expression of the corresponding ligand genes in the endometrium (x‐axis). Shown in (b) are all gene pairs in which average TPM was >2 for the receptor and > 1 for the ligand. The dashed lines indicate the cutoff for high expression [defined as TPM >10 (i.e., logTPM >1]. Gene pairs in which there was high expression of genes for both the receptor and its ligand were re‐graphed in (c) to facilitate identification of individual gene pairs

A total of 48 genes expressed in the morula were identified as encoding for receptors for growth factors or WNT ligands (Figure 2a). Of these, the 10 most highly‐expressed genes were *CD9* (a receptor for HBEGF), the adiponectin receptor *ADIPOR2*, the multifunctional integrin receptor component *ITGB1*, the nerve growth factor receptor *MAGED1*, the alternate dickkopf 1 receptor *CKAP4*, the adiponectin receptor *ADIPOR1*, the multifunctional receptors *SDC4* and *LRP2*, the WNT receptor *RYK*, and the PDGF receptor *PDGFRA*.

Expression of each receptor gene and the gene for the corresponding ligand by the endometrium are shown in Figure 2b. There were 35 instances where both the receptor gene in the morula and ligand gene in the endometrium were highly expressed (see Figure 2b upper right quadrant and Figure 2c). The receptors for the most highly‐expressed ligands were for midkine (*ITGB1, SDC4* and *LRP2*), *WNT5A* (*RYK*), the growth‐inhibitory protein *GAS6* (*MERTK*), the WNT agonist *NDP* (*LGR4*), *BMP4* (*ACVR2A* and *ACVR2B*), *VEGFA (ITGB1)*, and platelet‐derived growth factors (*PDGFRA*). Among the other receptors in this group were those for fibroblast growth factors (*SDC4* and *FGFR4*), DKK1 (*CKAP4*), transforming growth factor β proteins (*TGFBR1* and *SDC2*), and HGF (*SDC2*).

### 2.3 Genes encoding receptors for cytokines and chemokines

**Figure 3:**
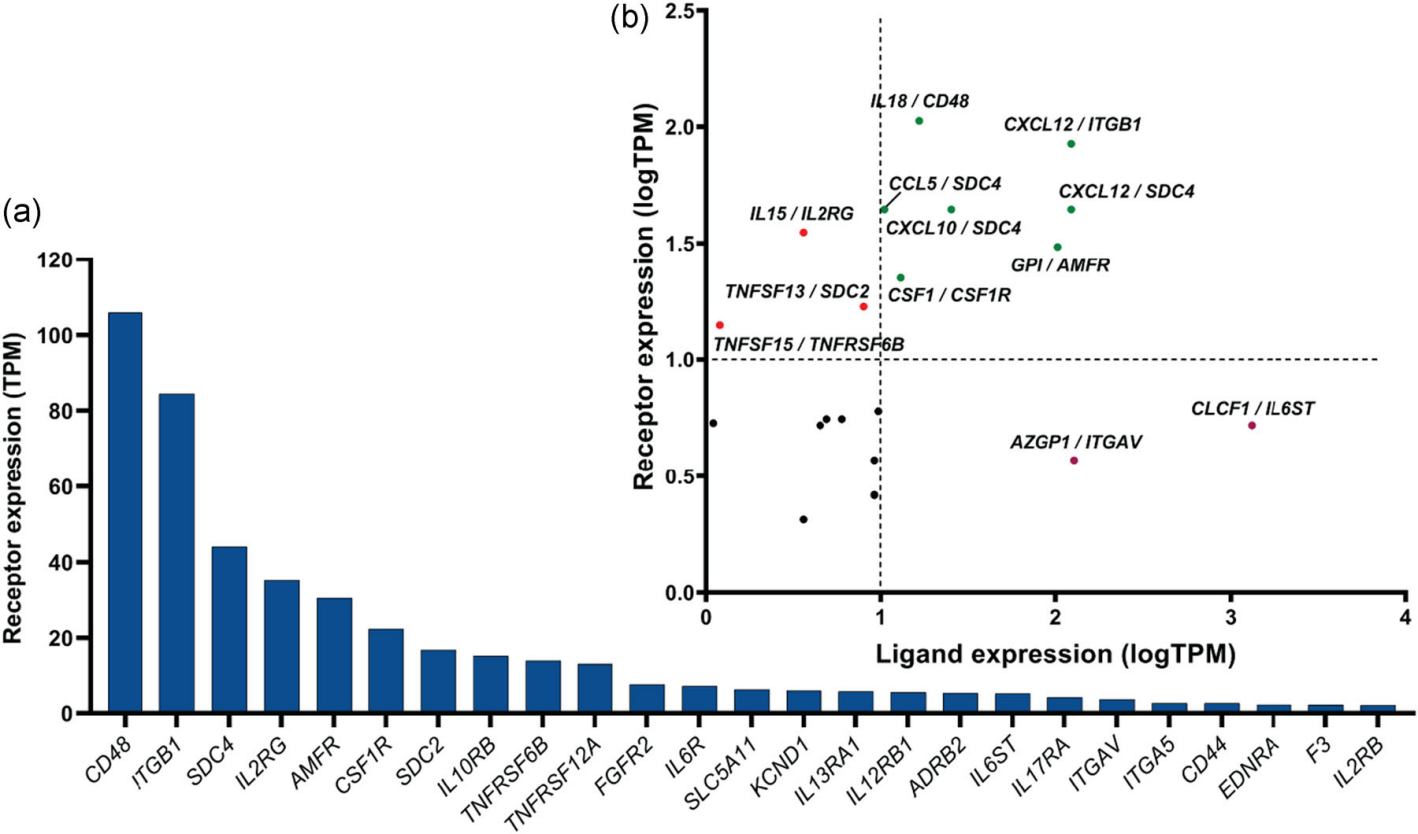
Relationship between the magnitude of expression by the bovine morula of genes encoding for receptors for cytokines and chemokines and expression of the corresponding ligand genes in the endometrium. (a) Transcript abundance for the 25 genes expressed by the morula that were characterized as encoding for receptors for growth factors or WNT ligands. Results are the average of 4 embryos. (b) Association between expression of individual receptor genes in the morula (y‐axis) and expression of the corresponding ligand genes in the endometrium (x‐axis). Shown are all gene pairs in which average TPM was >2 for the receptor and > 1 for the ligand. The dashed lines indicate the cutoff for high expression [defined as TPM >10 (i.e., logTPM >1]. Gene pairs in which there was high expression of genes for either the receptor, ligand, or both were identified individually

There were 25 genes expressed by the morula encoding for cytokine and chemokine receptors (Figure 3a), with the 10 most highly‐expressed receptor genes being *CD48* (receptor for IL18), the multifunctional receptors *ITGB1* and *SDC4*, *IL2RG* (receptor for several interleukins), *AMFR* (receptor for GPI), *CSF1R* (receptor for CSF1), *SDC2* (multifunctional), *IL10RB* (multifunctional receptor) and *TNFRSF6B* (receptor for TNFSF14, TNFSF15, and FASLG). There were seven instances where both the receptor gene in the morula and ligand gene in the endometrium were highly expressed (Figure 3b) including for the ligands *CXCL12* (receptors *ITGB1* and *SDC4*), the neurotrophic factor *GPI* (*AMFR*), *CXCL10* (*SDC4*), *IL18* (*CD48*), *CSF1* (*CSF1R*), and *CCL5* (*SDC4*) (Figure 3b).

### 2.4 Genes encoding receptors involved in juxtacrine and matricellular signaling

**Figure 4:**
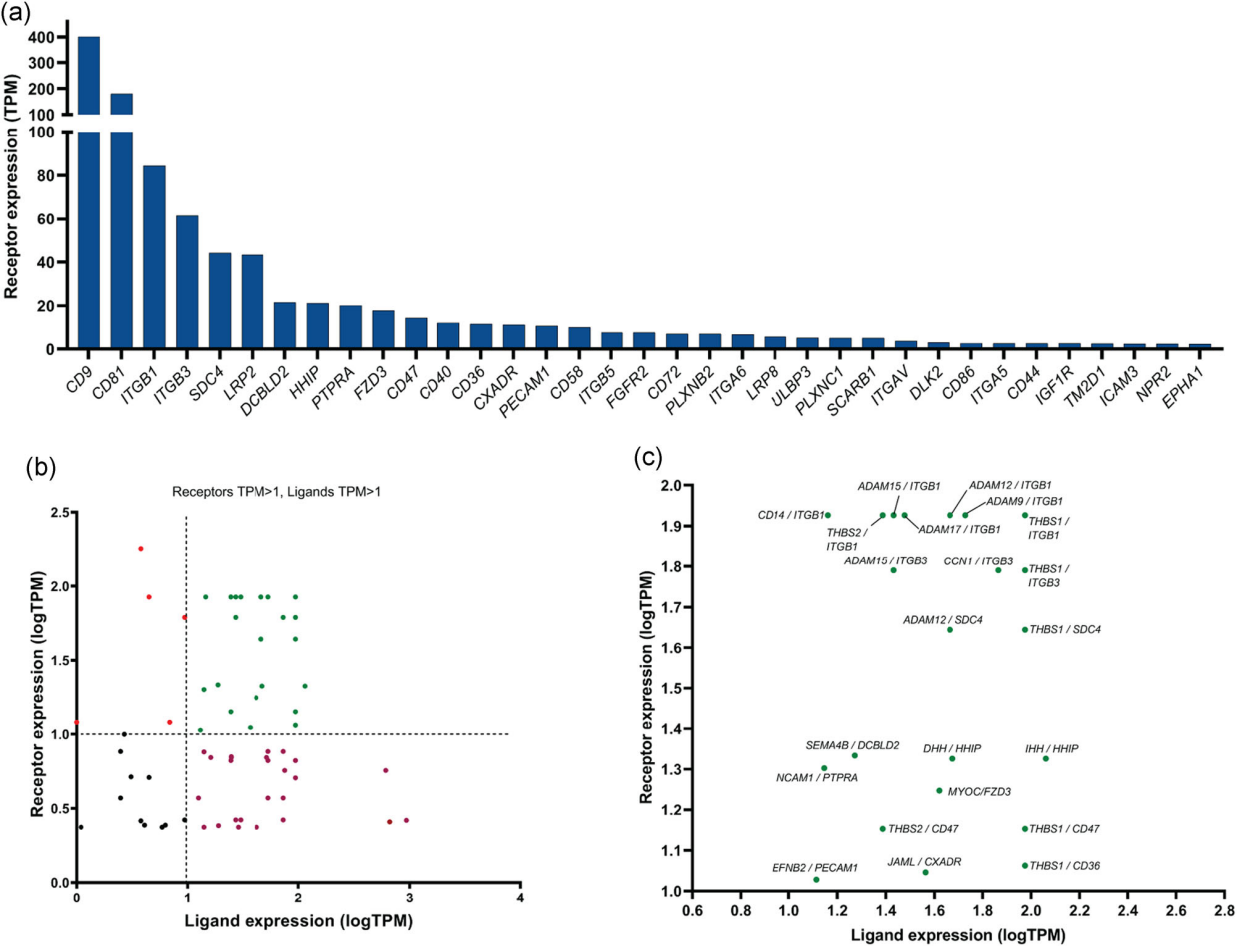
Relationship between the magnitude of expression by the bovine morula of genes encoding for receptors involved in juxtacrine and matricellular signaling and expression of the corresponding ligand genes in the endometrium. (a) Transcript abundance for the 35 genes expressed by the morula that were characterized as encoding for receptors involved in juxtacrine and matricellular signaling. Results are the average of 4 embryos. (b, c) Association between expression of individual receptor genes in the morula (y‐axis) and expression of the corresponding ligand genes in the endometrium (x‐axis). Shown in (b) are all gene pairs in which average TPM was >2 for the receptor and > 1 for the ligand. The dashed lines indicate the cutoff for high expression [defined as TPM >10 (i.e., logTPM >1]. Gene pairs in which there was high expression of genes for both the receptor and its ligand were re‐graphed in (c) to facilitate identification of individual gene pairs

A total of 35 genes expressed in the morula that encode for receptors involved in cell–cell or cell‐extracellular matrix signaling were analyzed for associated expression of ligand genes in the endometrium. The most highly expressed of the receptor genes was *CD9* (receptor for ADAM2 and HBEGF), *CD81* (receptor for complement C3 and GPC3), and the multifunction receptor components *ITGB1, ITGB3, SDC4*, and *LRP2* (Figure 4a). There were 22 ligand (endometrium) and receptor‐(embryo) pairs in which both genes were highly expressed: the thrombospondins *THBS1* and *THBS2* (*ITGB1*, *ITGB3*, *SDC4*, *CD47*, and *CD36*), hedgehog genes *IHH* and *DHH (HHIP*), *CCN1* (*ITGB3*), the ADAM metallopeptidase domain proteins *ADAM9* (*ITGB1*), *ADAM12* (*ITGB1* and *SDC4*), *ADAM15* (*ITGB1* and *ITGB3*), and *ADAM17* (*ITGB1*); the secreted glycoprotein *MYOC3* (*FZD3*); the junction adhesion protein *JAML* (*CXADR*), semaphorin *SEMA4B* (*DCBLD2*), *CD14* (*ITGB1*), *NCAM1* (*PTPRA*), and ephrin *EFNB2* (*PECAM*).

### 2.5 Receptors for small molecules

**Figure 5:**
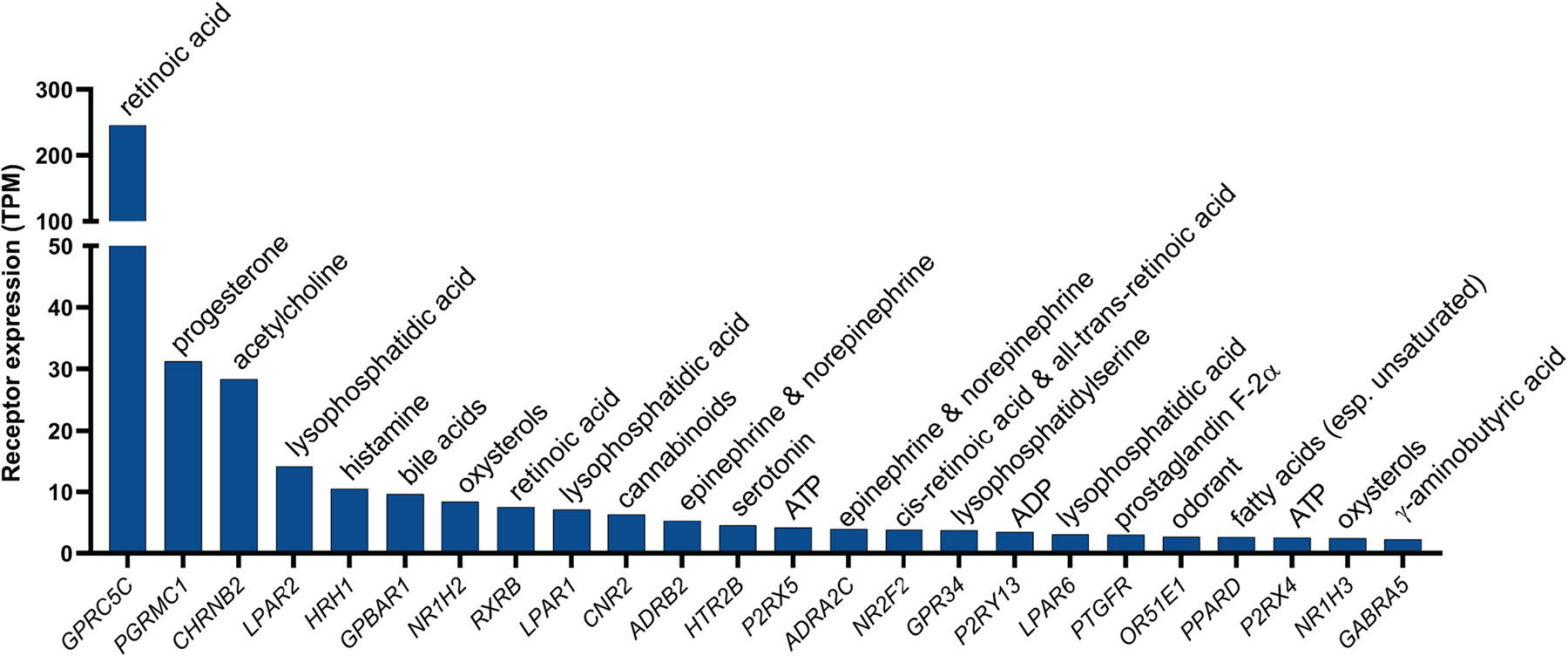
Transcript abundance in the bovine morula for receptor genes using small molecules as ligands. Results are the average of four embryos. Identification of the ligand for each receptor is indicated above the bars

There were 25 genes expressed in the morula for receptors of small molecule ligands (Figure 5). The most highly expressed were *GPRC5C* (retinoic acid receptor), *PGRMC1* (progesterone), *CHRNB2* (acetylcholine), *LPAR2* (lysophosphatidic acid), *HRH1* (histamine), *GPBAR1* (bile acids), *NR1H2* (oxysterols), and *ESRRG* (estradiol). Other notable receptors included *RXRB* (retinoic acid*), LPAR1, GPR34*, and *LPAR6* (lysophosphatidic acid), *CNR2* (cannabinoids) and *ADRB2* (catecholamines).

### 2.6 Ligand genes expressed in the morula

**Figure 6:**
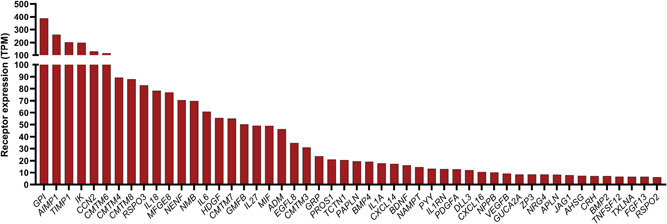
Transcript abundance in the bovine morula for the 50 most highly expressed genes encoding cell‐signaling ligands. Results are the average of 4 embryos

There were 84 genes identified as being expressed in the morula that encode for cell signaling ligands (Supporting Information File S1, Table S3). The proteins encoded by these genes could act either in autocrine regulation of the embryo or to regulate nearby endometrial tissues. Transcript abundance for the 50 most highly‐expressed genes are displayed in Figure 6. Among the top 10 expressed genes were 8 cytokine genes (*GPI, AIMP1, TIMP1, IK, CMTM6, CMTM4, CMTM8*, and *IL18*), the growth factor *CCN2* and the WNT‐regulatory molecule *RSPO3*.

Analysis of the ligand genes expressed in the morula by Ingenuity Pathway Analysis identified 85 canonical pathways in which there was overrepresentation (*p* < 0.05) of genes (Supporting Information File S1, Table S4). Among the pathways with at least 10 genes was IL‐17 signaling (*n* = 10 genes) and human embryonic stem cell pluripotency (*n* = 10 genes).

## 3 DISCUSSION

Once the embryo transits from the oviduct, the endometrium is the predominant tissue controlling the environment in which the embryo executes its developmental program. Examination of expression of genes for embryo receptors and their endometrial ligands has resulted in identification of putative cell signaling systems for embryo‐maternal communication that have not been previously suspected. In other cases, confirmatory evidence was provided for a biological role for specific cell‐signaling molecules previously reported to affect embryonic function. The atlas of embryo receptor gene and endometrial ligand gene expression should also be useful for understanding communication between the developing embryo and endometrium.

There are caveats inherent in interpreting data on gene expression. It is often the case that transcript abundance is not highly correlated with protein abundance. This is particularly so for the preimplantation embryo because of developmentally‐regulated changes in degradation of maternally‐derived messenger RNA (mRNA) and proteins, translation of maternal mRNA and embryonic gene expression (Sha et al., 2019; Toralova et al., 2020). It is also probable that some receptors that are present but not highly expressed could be important for embryo‐maternal communication, particularly if the gene was expressed earlier in development and the protein remains abundant in the embryo. One example might be for natriuretic peptide receptor 2 (*NPR2*). This gene was expressed by the morula (average TPM = 2.4) but at a lower level than for many other receptor genes. Nonetheless, treatment of bovine embryos with the prototypical ligand for NPR2, C‐natriuretic peptide, caused an increase in number of cells in the inner cell mass of the blastocyst (Sang et al., 2020). Furthermore, some endometrial genes could be expressed in compartments of the endometrium (stromal cells or endothelial cells) where secretion of the encoded protein or small molecule ligand into the uterine lumen might be impeded. Other ligands might be derived from the blood and not local synthesis. One of the most highly expressed receptor genes in the morula, for example, was *ADIPOR2*. Although the gene encoding its ligand, adiponectin, was nearly nondetectable in the endometrium, adiponectin is a hormone in peripheral circulation of both pregnant and nonpregnant cows (Urh et al., 2019). It is also pertinent to recognize that the current study was based on analysis of a small number of samples from specific types of animals. It is possible that there is significant variation in expression of specific genes among populations of embryos and females so that some ligand‐receptor pairs identified as highly expressed or lowly expressed might not always be so.

Identification of all the ligands produced by the endometrium that regulate embryonic function was also limited by incomplete knowledge of ligand usage by receptors. There was a total of 15 orphan receptor genes expressed by the embryo and it is probable that many other receptors have ligands that have not been described. Other modes of signaling such as via extracellular vesicles was not considered here but could be important for regulation of embryonic function (Bridi et al., 2020).

Among the growth factors and cytokines for which both the receptor and ligand gene were highly expressed are several that were previously not implicated in playing a role in regulation of the preimplantation embryo. Two of these endometrial ligands were originally described as neurotrophic molecules—midkine (*MDK*) and glucose‐6‐phosphate isomerase (*GPI*; also called autocrine motility factor). *GPI* was also highly expressed by the embryo. Three genes encoding for receptor proteins for MDK were highly expressed by the embryo (*ITGB1*, *SDC4*, and *LRP2*) and one receptor for GPI (*AMFR*) was highly expressed. The heparin‐binding growth factor MDK has been implicated in several functions potentially important for embryonic development including cell survival, differentiation, repair and migration (Muramatsu, 2014). Furthermore, GPI can promote cell motility and proliferation and block apoptosis (Nakajima & Raz, 2020). Two other ligand‐receptor pairs that were highly expressed and not previously implicated in embryonic development were the proinflammatory cytokine gene *IL18* and its receptor *CD48*, and growth arrest specific 6 (*GAS6*) and its receptor *MERTK*.

Genes for three chemokines not previously associated with embryonic development were also highly expressed in the endometrium: *CXCL12* (receptors *ITGB1* and *SDC4*), *CXCL10* (*SDC4*), and *CCL5* (*SDC4*). Thus, five of the previously unknown potential regulators of embryonic development (MDK, GPI, CXCL12, CXCL10, and CCL5) participate in cell migration. Another highly expressed ligand‐receptor pair was *WNT5A*‐*RYK*, which is also involved in cellular migration (Lin et al., 2010). Cell movement occurs in the preimplantation embryo, including transit of outer trophectoderm cells to the inner cell mass (Toyooka et al., 2016) and segregation of the inner cell mass into hypoblast and epiblast (Negrón‐Pérez et al., 2017).

There were also several highly‐expressed receptor‐ligand pairs demonstrated previously to modify development of the preimplantation embryo. Of these, ligands shown to have positive effects on development of the bovine embryo include PDGF (Lim & Hansel, 1996; Yang et al., 1993) and transforming growth factor‐β (Lim & Hansel, 1996; Neira et al., 2010; Yang et al., 1993). Two other ligands, VEGFA (Biswas et al., 2018) and CSF1 (Bhatnagar et al., 1995), have been reported to have positive effects on development of the embryo in other mammalian species. In contrast, two other ligands encoded by highly‐expressed genes, BMP4 and HGF, reduced development of the bovine embryo (Kannampuzha‐Francis et al., 2017; Sang et al., 2020).

Several molecules involved in WNT signaling were highlighted in the current datasets. WNT can activate several signaling pathways and there is evidence that inhibition of the canonical or β‐catenin‐dependent WNT signaling pathway may be important for development of the bovine blastocyst. Pharmacological activation of canonical WNT signaling reduces the percent of bovine embryos developing to the blastocyst stage (Aparicio et al., 2010; Denicol et al., 2013; Tríbulo et al., 2017). Moreover, maintenance of cells of the inner cell mass in a pluripotent state is facilitated by inhibition of WNT signaling (Xiao et al., 2021). Among the members of the WNT signaling system present in the bovine embryo and endometrium were the canonical WNT ligands *NDP*, expressed by the endometrium in conjunction with high expression of its receptor *LGR4* in the embryo, and *RSPO3*, which was expressed by the embryo. Another WNT ligand‐receptor pair that was highly expressed, *WNT5A‐RYK*, can activate the WNT/Ca^2+^ and planar‐cell polarity pathways and either activate or inhibit canonical signaling (Clark et al., 2014; Roy et al., 2018). There was also high expression of the canonical WNT antagonist *DKK1* by both the endometrium and embryo. Treatment of bovine embryos with DKK1 can block the inhibitory actions of canonical WNT activators, increase TE formation, trophoblast elongation and competence of embryos to survive to term after transfer into recipients (Denicol et al., 2013, 2014; Tríbulo et al., 2017, 2019). The most highly expressed receptor for DKK1 in the embryo was *CKAP4*, which signals through AKT to increase cell proliferation (Kikuchi et al., 2017).

Several receptors for small molecules were highly expressed in the embryo. There is nothing known about the role of some of these receptors or their ligands in the preimplantation embryo. This is the case for the acetylcholine receptor *CHRNB2* and acetylcholine, the ATP receptors *P2RX5* and *P2RX4*, the ADP receptor *P2RY13*, the odorant receptor *OR51E1*, the fatty acid‐activated transcription factor *PPARD*, and the γ‐aminobutyric acid receptor *GABRA5*. There was low transcript abundance in the endometrium for the enzyme to synthesize acetylcholine, (*ACHE*) and CHRNB2 may have another unknown ligand besides acetylcholine. One of the most highly‐expressed genes encoding receptors for small molecules was *PGRMC1* but its ligand, progesterone, was without effect on development of the preimplantation bovine embryo in vitro (Clemente et al., 2009; Larson et al., 2011) or in vivo (Carter et al., 2010). Perhaps, progesterone has actions on the embryo that were not examined in these papers or PGRMC1 has additional ligands besides progesterone that are important for regulating the embryo.

Other ligands for receptor genes recognizing small molecules have been reported to have actions facilitative for embryonic development and survival. For example, treatment of bovine embryos with a retinoic acid receptor agonist increased the proportion of morulae developing to the blastocyst stage (Gómez et al., 2008). Lysophosphatidic acid has been shown to change gene expression of the bovine embryo (Torres et al., 2014) and hasten blastocoel formation and decrease apoptosis in the pig embryo (Shin et al., 2018). Another example is for the oxysterol receptor *NR1H2* which functions to prevent cholesterol overload in membranes (Xiao et al., 2010). Treatment of mouse embryos with 7‐ketocholesterol reduced development in a cholesterol‐dependent manner (Pratt et al., 1980). Another gene expressed in the embryo, *GBAR1*, encodes for a receptor activated by the bile acid tauroursodeoxycholic acid to stimulate DNA repair in the pig embryo and reduce the negative effect of ultraviolet light on development to the blastocyst stage (Dicks et al., 2020). Histamine has been implicated in synergizing with prostaglandins to facilitate implantation in the mouse, rat and rabbit (Cikos et al., 2011).

Other small molecule ligands have been reported to be inhibitory to preimplantation development including adrenergic receptor agonists (Cikos et al., 2005), serotonin (the ligand for HTR2B) (Veselá et al., 2003) and prostaglandin F‐2α (the ligand for PTGFR) (Scenna et al., 2004; Kim et al., 2014). Another receptor gene expressed by the embryo (*CNR2*) binds endogenous cannabinoids. Exposure of 2‐cell mouse embryos to the endogenous cannabinoid anandamide reduced development in 2‐cell mouse embryos (Paria et al., 1998) and induced apoptosis in hatched sheep blastocysts (Turco et al., 2008); these effects were mediated by CNR1 and not CNR2, however.

Communication between the preimplantation embryo and underlying endometrium is important for optimizing competence of the embryo for survival later in development (Denicol et al., 2014; Loureiro et al., 2009). This communication is not unidirectional but rather the embryo, at least from the blastocyst stage onwards, can also modify secretory activity of the uterine endometrium (Sponchiado et al., 2017, 2019). Identification of the receptor and ligand genes described in this paper will provide direction for further experimentation to unraveling communication networks between the embryo and endometrium.

## 4 MATERIALS AND METHODS

### 4.1 Datasets for gene expression in the morula and endometrium

An RNA‐Seq dataset of gene expression of the bovine in vivo derived morula was obtained from Jiang et al. (2014). Data were from a total of four morulae recovered from superovulated cows—two early morulae collected at Day 5 after estrus and two compact morulae collected at Day 6 after estrus. Individual embryos were subjected to RNA extraction, linear amplification, and RNA‐Seq as detailed elsewhere (Jiang et al., 2014). Reads were mapped to bovine reference genome UMD3.1.1 using HISAT2 version 2.0.5 (https://daehwankimlab.github.io/hisat2/). The raw FASTQ files and normalized read counts per gene are available at Gene Expression Omnibus (GEO) (http://www.ncbi.nlm.nih.gov/geo) under the accession number GSE59186. For this paper, gene expression was quantified as TPM using isoEM (https://dna.engr.uconn.edu/). Data for TPM were averaged for the four embryos. Data for TPM of all genes are presented in Supporting Information File S1, Table S5.

The RNA‐Seq dataset for endometrium was obtained from Sánchez et al. (2019b). Data were from a total of five individual samples of endometrium collected from the uterus of cyclic heifers at Day 5 after estrus. Endometrium was collected from the uterine horn ipsilateral to the side of the corpus luteum. RNA was extracted and RNA‐Seq performed as described (Sánchez et al., 2019b). Data on read counts per gene were downloaded from the GEO database at https://www.ncbi.nlm.nih.gov/geo/(GSE118507). Reads were converted to TPM using StringTie v2.1.1 at https://ccb.jhu.edu/software/stringtie. Values for TPM were averaged for the four heifers. Data for TPM of all genes are presented in Supporting Information File S1, Table S6.

### 4.2 Identification of embryo receptor genes and genes associated with the corresponding ligands

Receptor genes expressed by the morula were identified by submitting the list of genes having average TPM >2 to Ingenuity Pathway Analysis (Qiagen) and selecting genes encoding for proteins categorized as G‐protein coupled receptors, ligand‐dependent nuclear receptors or transmembrane receptors. Additionally, other receptor genes were identified by comparing the list of expressed genes with average TPM >2 to the list of receptor genes compiled by FANTOM5 Project atlas of human ligand‐receptor pairs (Ramilowski et al., 2015). Genes were then scrutinized individually to remove those encoding for proteins that were not considered functional receptors. The final number of receptor genes was 175 (Supporting Information File S1, Table S1).

Ligands for each receptor expressed in the morula were identified using the FANTOM5 Project atlas (Ramilowski et al., 2015), GeneCards (www.genecards.org) (Stelzer et al., 2016) and Wikipedia (wikipedia.org). Information for other ligand‐receptor pairs was obtained from the primary literature; references are included in Supporting Information File S1, Table S1. Relevant endometrial genes included genes encoding protein ligands as well as selected enzymes involved in synthesis of nonprotein ligands. Canonical pathways in which morula receptor and morula ligand genes were overrepresented were determined by Ingenuity Pathway Analysis.

Data on expression of morula receptor and ligand genes were used to construct graphs to show relative abundance of mostly‐highly expressed genes in each class. Furthermore, expression of each receptor gene was related to endometrial expression of the corresponding ligand gene by constructing a table of expression values (Supporting Information File S1, Table S1) and generating graphs that illustrated expression of individual receptor genes and the corresponding ligand genes.

## CONFLICT OF INTERESTS

The authors declare that there are no conflict of interests.

## Supporting information

Supporting information.

## Data Availability

The data that supports the findings of this study are available in the Supporting Information material of this article.
